# Using contribution analysis to evaluate health professions and health sciences programs

**DOI:** 10.3389/fmed.2023.1146832

**Published:** 2023-10-02

**Authors:** Tammie Choi, Mahbub Sarkar, Maxine Bonham, Tina Brock, Ingrid Ann Brooks, Basia Diug, Dragan Ilic, Arunaz Kumar, Wee-Ming Lau, Jennifer Lindley, Julia Morphet, Margaret Simmons, Evelyn Volders, Paul J. White, Caroline Wright, Claire Palermo

**Affiliations:** ^1^Monash Centre for Scholarship in Health Education, Monash University, Melbourne, VIC, Australia; ^2^Department of Nutrition, Dietetics and Food, Monash University, Melbourne, VIC, Australia; ^3^Faculty of Pharmacy and Pharmaceutical Sciences, Monash University, Melbourne, VIC, Australia; ^4^School of Nursing and Midwifery, Monash University, Melbourne, VIC, Australia; ^5^School of Public Health and Preventive Medicine, Monash University, Melbourne, VIC, Australia; ^6^Department of Obstetrics and Gynaecology, School of Clinical Sciences, Monash University, Melbourne, VIC, Australia; ^7^Jeffrey Cheah School of Medicine and Health Sciences, Monash University Malaysia, Subang Jaya, Selangor, Malaysia; ^8^Monash Rural Health, Monash University, Melbourne, VIC, Australia; ^9^Department of Medical Imaging and Radiation Sciences, School of Primary and Allied Health, Monash University, Melbourne, VIC, Australia

**Keywords:** contribution analysis, curriculum evaluation, learning and teaching, practice ready, health professions, health science

## Abstract

**Introduction/background:**

Course evaluation in health education is a common practice yet few comprehensive evaluations of health education exist that measure the impact and outcomes these programs have on developing health graduate capabilities.

**Aim/objectives:**

To explore how curricula contribute to health graduate capabilities and what factors contribute to the development of these capabilities.

**Methods:**

Using contribution analysis evaluation, a six-step iterative process, key stakeholders in the six selected courses were engaged in an iterative theory-driven evaluation. The researchers collectively developed a postulated theory-of-change. Then evidence from existing relevant documents were extracted using documentary analysis. Collated findings were presented to academic staff, industry representatives and graduates, where additional data was sought through focus group discussions - one for each discipline. The focus group data were used to validate the theory-of-change. Data analysis was conducted iteratively, refining the theory of change from one course to the next.

**Results:**

The complexity in teaching and learning, contributed by human, organizational and curriculum factors was highlighted. Advances in knowledge, skills, attitudes and graduate capabilities are non-linear and integrated into curriculum. Work integrated learning significantly contributes to knowledge consolidation and forming professional identities for health professional courses. Workplace culture and educators’ passion impact on the quality of teaching and learning yet are rarely considered as evidence of impact.

**Discussion:**

Capturing the episodic and contextual learning moments is important to describe success and for reflection for improvement. Evidence of impact of elements of courses on future graduate capabilities was limited with the focus of evaluation data on satisfaction.

**Conclusion:**

Contribution analysis has been a useful evaluation method to explore the complexity of the factors in learning and teaching that influence graduate capabilities in health-related courses.

## Introduction

Evaluation plays an important role in education ensuring the quality and impact of teaching and learning. More specifically, in health professions and health sciences education, evaluation contributes to ensuring that desired graduate outcomes fulfil community health needs and assure health regulatory authorities, educational regulators, employers and patients/clients that programs of study produce safe, professional, effective and work-ready and fit-for-purpose practitioners (1). Additionally, evaluation can be a tool for assessing curricular relevance, satisfying learner needs, assessing the constructive alignment with institutional standards (2), and maximizing instructional resources (3). Despite the recognized need for rigorous evaluation of health profession’s education curricula (3), there are few published examples evaluating health curricula in the literature (4–6).

Current approaches focus on the evaluation of individual concepts, disciplines or content, such as evidence-based medicine (5) or preparation for practice (4, 6). These approaches provide evidence for student satisfaction and acquisition of knowledge and skills, with many using the Kirkpatrick’s (7) model of program evaluation (8). However, the Kirkpatrick’s model has been recently criticized for not capturing the full impact of learning and development (8). This criticism is, in part, because of a focus on process evaluation measures, such as appropriateness, satisfaction, numbers enrolled, demography, and the assessment of knowledge and skills (8). Such evaluation models might not sufficiently explore the complexity of the factors (e.g., learning, teaching, assessment, research, healthcare delivery, community engagement and settings of health care programs) that lead to different outcomes in preparing health professionals for practice (9, 10). Due to the complexity of health professions education programs, few evaluation strategies have the capacity to holistically consider student behavior change (both personal and professional development) or graduate outcomes (11). Such factors are often considered beyond the scope of a single health professions course.

Traditionally, attribution analysis (AA) has been used to determine whether the curricular outcomes being studied are attributable to the program, i.e., did the program cause the observed outcomes. AA is a positivist-orientated approach assuming a unidirectional causal relationship and focusing on short-term outcomes (10). In the complex system of teaching and learning where learning is caught not taught (12), the linearity of this evaluation approach has been widely criticized (13). It would be beneficial for educators to consider alternative approaches.

Contribution analysis has been proposed as an alternative approach to evaluate health professions education. Contribution analysis (CA) aims to explore how and why various elements of a program contribute to the outcomes of interest (10), e.g., how the professional accreditation standards contribute to the observed outcomes. By collecting information from multiple sources (e.g., documents and interviews), CA uses an expert-derived theory of change (14, 15) to explore the interactions between program and curricular activities and connect their relationship to proximal (program-related outcomes) and distal outcomes (system-level outcomes), and the assumptions informing these connections (10). CA aligns with contemporary recommendations of health professions education program evaluation to comprehensively capture contributing factors and the emergent processes toward development of the outcomes of interest (1). While CA has been used extensively to evaluate complex public health and health promotion interventions (16), there is a paucity of research using CA as an approach for the evaluation of health professions education curricula (10, 17). To the authors’ knowledge there have not been any published examples of the use of CA as an evaluation approach in health professions education.

Our study aimed to evaluate health professions and health sciences programs using Contribution Analysis. Specifically, we are exploring what factors support and hinder teaching and learning and what teaching and learning factors contribute to the development of fit-for-purpose health profession graduates. The identification of these factors and their relationships will inform a more holistic evaluation approach of health professions and health sciences programs and enhance outcome-directed teaching.

## Methods

### Design

This study applied Mayne’s six-step contribution analysis (15, 18) to evaluate the outcomes of a convenience sample of six health professions/health science programs offered at a large Australian university (name removed for peer review). In our study, we applied CA to identify relevant health professions graduate outcomes and develop a theory of how and why factors that have contributed to this outcome (10, 14, 15). Utilizing CA approach allowed us to describe the complex pathways learners experience as they move toward these outcomes and explore the relationships between the different contributing factors to the outcomes (10). An important aspect of our study was to clearly define the terminology including ‘outcomes’, or the attributes of learners who are competent to practice. We also explicitly identified the assumptions regarding cause and effect and the theory behind these assumptions. We then used existing data to build a model that illustrated the relationships between external influences, outcomes, results (or processes that have led to impact), assumptions and risks. We took a pragmatic approach and we did what was possible and potentially feasible to allow replication.

### Sampling

The study was conducted across four health professions and two health sciences education programs at two faculties - Faculty of Medicine, Nursing and Health Sciences and Faculty of Pharmacy and Pharmaceutical Sciences at a large metropolitan university in Australia. Collectively these faculties offer five different undergraduate health sciences and 12 health professions entry-to-practice programs (post- and undergraduate level). The programs chosen for evaluation conveniently elected to participate in this evaluation and included four health professional (medicine, nursing, dietetics and pharmacy) and two health science (nutrition science and health science) programs with a total number of graduating students approximately 1,400 per year across the programs.

Our research team comprised 16 members, many of whom had a large stake in the project as program coordinators of the above programs. The team was diverse in terms of demographics (e.g., age, gender), disciplinary background, geographical location, teaching, research, as well as different levels of orientation and expertise with qualitative and quantitative research. At the beginning of the project, we undertook a team reflexivity exercise (19) with all authors, where discussion was facilitated by CP to acknowledge, reflect on and understand our own power and positioning at both an individual and system level as educators. It provided us with a valuable opportunity to understand each other’s perspectives, as well as cultivating a collaborative and rigorous approach to the analysis and interpretation of cross-disciplinary data. The reflexivity exercise identified that the team was motivated by outcomes and learnings about the impact of curricula on graduate outcomes. This exercise also provided a platform to share teaching experiences and curricular resources.

### Data collection and analysis

The six-step iterative process involved a series of data collection and analysis processes.

**Figure 1:**
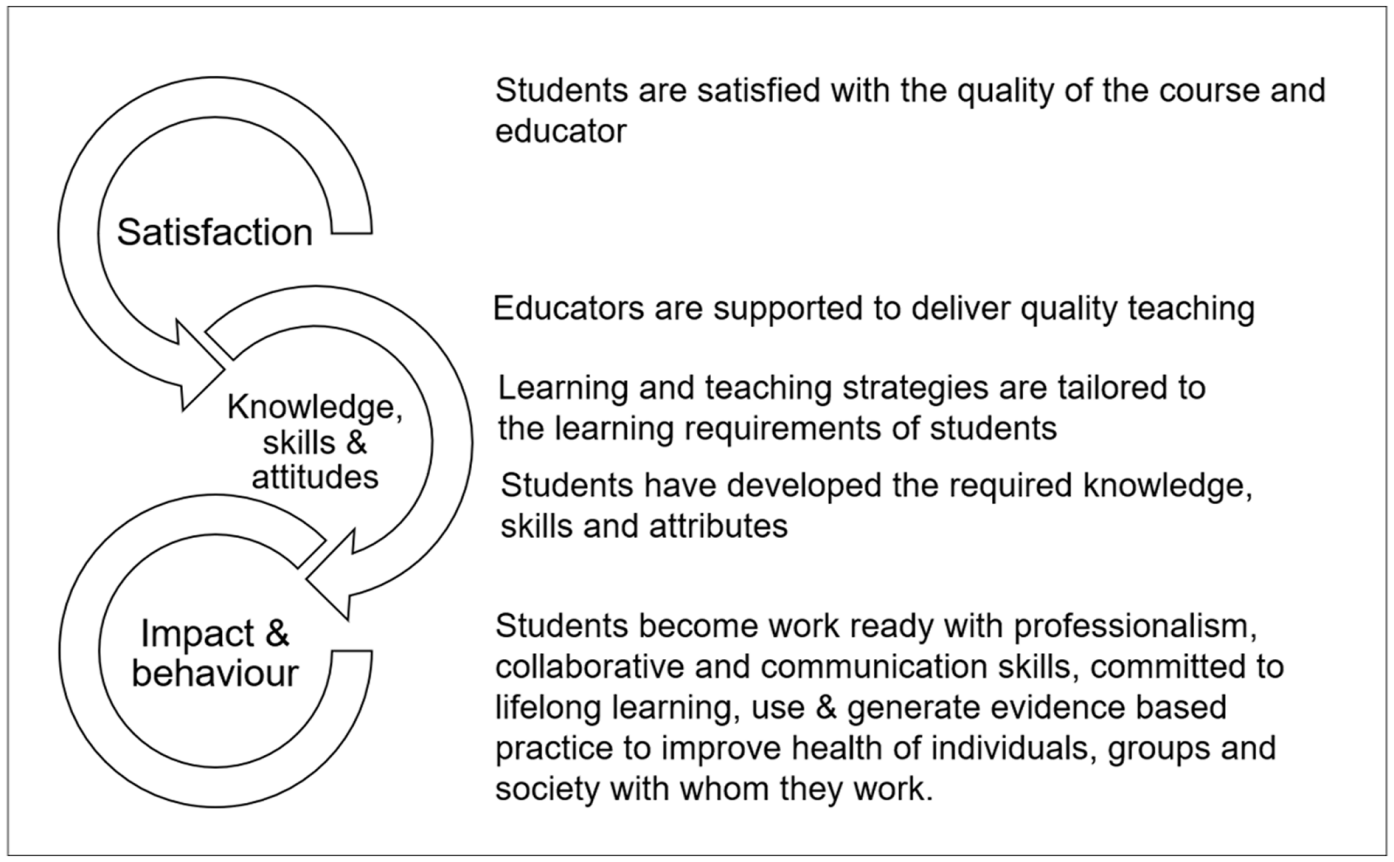
Program evaluation framework.

*Steps 1 (Develop the results chain) and 2 (Assess the existing evidence on results):* CA’s first step is to set out the attribution problem to be addressed. In our study, this happened in two stages. The first was identification of the health professions and health science education programs that would allow us to understand the processes of graduate outcome development in different teaching and learning contexts within health-related disciplines, and the second was identifying the nested theories of change in these education programs. The latter was done after partial implementation of CA Step 2 where the initial theory of change was established by building on a previously developed program evaluation framework (Figure 1). This program evaluation framework was developed prior to the commencement of the study by the Faculty Evaluation Strategy Working Group, which included a subset of the research team. The framework postulated that: if students are satisfied with the program and educators, and educators are supported to deliver quality tailored teaching (i.e., proximal shorter-term outcomes), students will develop the knowledge, skills and capabilities required to work as health professionals in practice (i.e., distal longer-term outcomes). We hereafter refer to these distal longer-term outcomes as graduate outcomes.

The framework included six common graduate outcomes as identified through a content analysis approach across the competency standards of 12 vocational health professional courses, including medicine (20), nursing (21), dietetics (22) and pharmacy (23). These common graduate outcomes were: (1) collaborate and work effectively in teams, (2) commit to lifelong learning, (3) demonstrate effective communication skills, (4) use and generate evidence; (5) improve health and (6) display professionalism in their practice. The assumption underpinning the evaluation was that graduates of the included courses achieve these graduate outcomes as evidenced by their current accreditation status. Thus, the evaluation did not set out to determine if these outcomes were achieved, but rather focused on the contributing factors that led to these outcomes.

**Figure 2:**
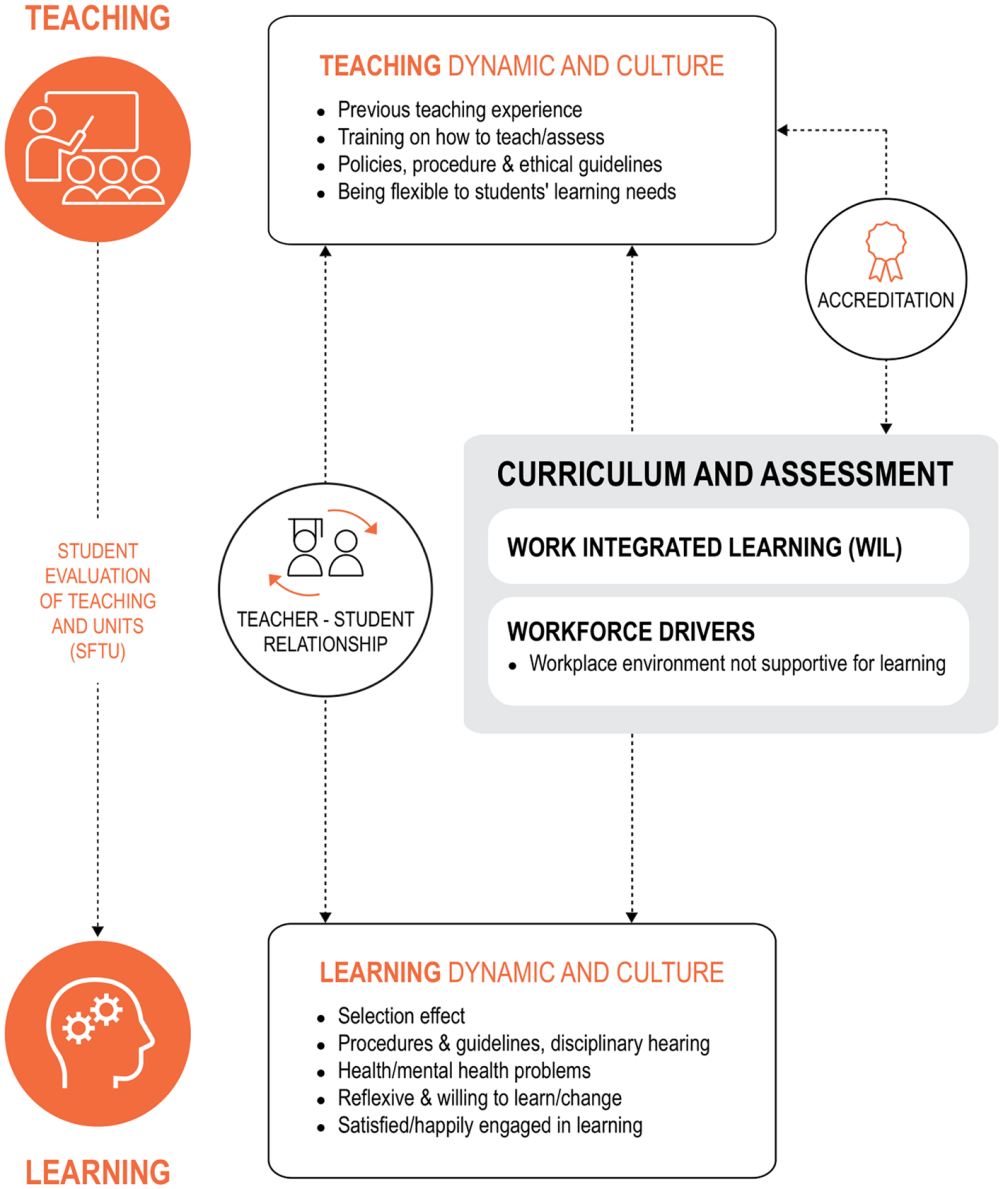
Initial logic model of factors influencing the achievement of graduate outcomes.

During step 2, senior academic educators (authors of this paper) from each education program were invited to contribute to the postulated theory of change (TOC), i.e., what contributes toward the graduate outcomes of interest within their program. The TOC represents hypothesized cause and effect relationships and related assumptions within an overall pathway of change, contributing to proximal and distal outcomes indicated in the program evaluation framework (Figure 1). Broad factors affecting graduate outcomes were identified as ‘human’, ‘organizational’ and ‘curricula’. Human factors were defined as the skills, qualities and personal education philosophy of the academics, work-based learning educators/clinical teachers, students and patients/communities. Organizational factors were acknowledged as the university and policy-related issues that impact on curriculum and outcomes. Examples of organizational factors include assessment policy, student discipline procedures, human resources policy and processes, resources provided for teaching and learning and work-based learning organizational cultures. Curricular factors included content, pedagogical design and quality of delivery. In addition, the role of accreditation in influencing curriculum was identified. In developing the initial TOC, the research team explicitly acknowledged related assumptions influencing the learning and teaching process, including previous learning and teaching experiences shaping individual student or teacher education expectations, individual educators’ pedagogical methods, educator-student relationships, health and mental health wellbeing of students and teachers, impact of disciplinary hearing on learning behaviors, and measurement of teaching quality. From the discussion, the initial logic model (Figure 2) was developed.

*Step 3 (Assess the alternative explanations):* All authors identified and gathered all potential sources of existing evaluation data to test the TOC and provide contextual insights. Relevance was the core criterion for all sources of evidence. These sources of data were grouped under four main areas: (i) relevant accreditation documentation; (ii) program materials; (iii) teaching evaluations and (iv) student demographics and completion data (Table 1). Seeking diverse sources of evidence respected the inherent complexity of learning and teaching. Additional opportunistic interviews with student discipline officers were conducted to gather insights about student discipline procedures which were the same across the two faculties.

**Table 1:** Description of sources of existing evaluation data.

Type of information source	Description of information source
Accreditation documentation	The most recent accreditation reports with appendices were obtained from each program, ranging from an 84-page report with 68 appendix items to a 537-page report. At the time of study, the Pharmacy, Nursing and Health Sciences programs were undergoing a revision and did not have accreditation documents. Internal and external program review reports were included for analysis instead.
Program materials	Unit guides (subject outlines) of all units in the included programs were obtained from the university unit guide portal. Specifically, the unit objectives and relevant assessment tasks were extracted and mapped against the six graduate outcomes.
Teaching evaluation	Anonymous Student Evaluation of Teaching and Units (SETU) responses of individual units were collated to provide a broad indicator of students’ satisfaction with teaching and the students’ learning journeys.
Student demographic and completion data	Class size, completion rate and student data including gender, age, Australian citizenship status, country of birth, socioeconomic status and geographical classification (metropolitan and regional) were extracted and added to the contribution story.

*Step 4 (Assemble the performance story):* The existing data were assembled and assessed by TC for its strengths and limitations. Document analysis was employed to extract relevant data for building the initial contribution story. Specifically, the process involved collating student demographic information, academic staff qualifications, student evaluations of each unit, mapping learning objectives of individual unit of each course against the six graduate outcomes, and extracting information from the accreditation reports and assessment policies to explain the contribution of human, organizational and curricular factors to the development of the graduate outcomes.

*Steps 5 (Seek out additional evidence) and 6 (Revise and strengthen the performance story):* The gathered evidence of each course was used to iteratively refine the initial Contribution Story. The refined Contribution were presented for assessment by stakeholders *via* a focus group at respective stage of data collection. These stakeholders were identified as academic staff, clinicians involved in work-based placements/learning, employers of graduates and graduates of the programs themselves. Invitations to all current academic staff were sent *via* program coordinators and a convenience sample of participants of work-based placement educators, employers and graduates volunteered to participate in the focus groups. A total of 44 participants took part in six focus groups (Table 2) which lasted between 65 and 124 min. Altogether we collected 512 min of audio data.

**Table 2:** Included documents and focus group participants from each program.

	Nutrition science	Dietetics	Nursing*	Pharmacy*	Medicine	Health sciences*
Documents reviewed						
Student demographics	√	√	√	√	√	√
Accreditation report	√	√			√	
Unit guides	√	√	√	√	√	√
SETU	√	√	√	√	√	√
Placement supervisor & student survey	-	√	-	-	-	-
Faculty program review with unit map	-	-	√	√	-	-
Class activity evaluation	-	-	-	-	√	-
Program documents	-	-	-	-	-	√
Focus group participants (n)						
Academics	3	7	4	6	4	4
Work based learning educators	2	-	2	3	4	2
Graduates	2	-	-	-	1	-
Total	**7**	**7**	**6**	**9**	**9**	**6**

SETU, Student Evaluation of Teaching and Units. *New curriculum, no graduates yet.

These focus groups were designed to assess the existing evidence and strengthen the contribution story. See questions in Table 3. As such, the focus groups involved a program-specific presentation on summarized existing data followed by a set of structured questions. The questions explored whether participants perceived the data to be reflective of teaching and learning in their relevant program, if any key data sources were missing from the summaries, whether the program was effective in facilitating student development of the six graduate outcomes and if the program developed any other competencies. This stage of evidence assessment was done iteratively where the Contribution Story was strengthened and revised after each focus group. The iterations helped to uncover a small number of additional data sources, including evaluation reports of teaching activities and notes from curricular design planning. The iterations also helped the research team experiment with different data displays to examine the strengths and limitations of the contribution stories.

**Table 3:** Guided focus group questions.

	Question
1	Drawing on your experience as an educator or past student, do you think the presented analysis captures what’s happening in your course in terms of teaching & learning? In area of student satisfaction, knowledge skills & attitude development, and impact & behavior
2	Do you think we have missed any key data / document that could tell us more about your course?
3	Specifically, do you think your course is effective in facilitating student development in the core capability areas (teamwork, lifelong learning, effective communication, evidence-based practice, improving health & professionalism)?
4	Do you think there is desirable quality or capability required as a health professional not captured in our analysis?
5	If you can provide improvement suggestion to any part of training health professionals, what would that be & why?

Focus groups were audio-recorded and recordings transcribed verbatim and checked for accuracy by respective focus group facilitator (TC, CP, or MS). In order to develop a deeper understanding of the qualitative data, the transcripts were read several times by the first author (TC) (24). Focus group transcripts were analyzed thematically using an iterative coding process, whereby all transcripts were coded line by line inductively before grouping codes into similar concepts or themes selected to answer the evaluation questions for the study (25). To ensure robustness of data analysis, analysis of all transcripts was first undertaken by TC followed by a second author (CP or MS). Both authors came together to discuss their independent findings and, in most cases, came to consensus. A third author (CP or MS) was involved in the discussion where the two authors who completed the analysis could not come to a consensus in the first instance.

Results from the thematic analysis of focus group transcripts were triangulated with document analysis. The themes were then utilized to build the Contribution Story iteratively (10). In this approach, steps three to six were an ongoing cycle from program to program, allowing us to keep refining and strengthening our final Contribution Story patterns common across all programs. As such, each subsequent focus group was presented with an adapted version of the contribution story. The focus group discussion helped to enhance our contextual understanding of the relationship between learning and teaching activities and proximal outcomes of learner satisfaction and distal outcomes of development of graduate outcomes. The themes identified from the focus group data described below were used to inform the Contribution Story. To ensure anonymity of participants, we intentionally avoided naming the discipline and role of the person who expressed the quotes.

## Results

The six focus groups with academic staff, clinicians, employers and graduates provided in-depth contextual information to the collated input from the variety of evidence. They shared experience and expert opinion on factors supporting and hindering teaching and learning, and what teaching and learning factors contribute to the development of competent health profession graduates. The shared narratives provided contextual information and insight of teaching and learning that helped to refine and strengthen the logic model toward development of the final Contribution Story. Four themes were identified from the focus groups. They described learning and teaching strategies to enhance development of the graduate outcomes of interest. Only quotes from the focus groups were used in presentation of the findings as it was the final step of contribution analysis and the focus group participants added contexts to the gathered evidence in earlier steps.

### Theme 1: the need for explicitly described learning objectives in curricula

Focus group participants reported incorporation of the development of the six competencies of interest (working collaboratively in a team, effective communication, lifelong learning, evidence-based practice, professionalism, improving health) in respective curricula. However these competencies were often implied in the wordings of unit guides and hidden within accreditation-body-approved curriculum. It was highlighted during our document analysis that teaching and facilitation of development of these student behavioral competencies were often implicit, rather than explicit. During focus groups, participants described teaching, role-modeling, designing authentic assessment tasks (e.g., group presentations, critical essays) and Work Integrated Learning (WIL) as vehicles to promote development of the behavioral competencies. However, such vehicles were not explicitly communicated in unit guides and assessment instructions. One educator commented on the ambiguities between competencies required and learning outcomes:

“…[w]e teach them critical thinking and reflective [thinking] but it’s not actually mentioned [in the unit objectives]. I don’t know if you can always teach critical thinking. I think it’s almost a common sense.” (Focus group 1, participant A)

The lack of explicitly described learning goals for teaching and assessments was reported to contribute to inconsistency in teaching delivery. The high percentage of sessional staff (i.e., part-time instructors) and/or turnover of teaching staff across all programs, and their varied educational qualifications and teaching experience, were reported to be a key concern regarding achievement of learning outcomes:

“…[d]ifferent people interpret things [in the unit guide] differently.” (Focus group 2, participant A)

### Theme 2: recognizing learning priorities and challenges in work integrated learning

Work Integrated Learning (WIL) provides a unique training environment and a platform for teachable moments, consolidating knowledge learnt in the classroom to application into practice. Teaching and learning was explained as a fluid process and highly influenced by qualities of teachers and students, and contextual circumstances during the process. Captured in student feedback within accreditation reports, the WIL setting was reported to be an unstructured environment for learning, which could further challenge consistency in the education experience for students. This variability made teachable moments unplanned, opportunistic and episodic, involving field-educators and, sometimes, patients:

“…[m]y unit is set up so theoretically all this stuff would happen. But I’m not sure if it does in real life, and does consistently across all the placement sites.” (Focus group 3, Participant A)

In the fast and complex clinical environment, errors and complaints are unavoidable. Educators and teachers reported capitalizing on such critical incidents in healthcare and made these teachable moments. Incidental teaching moments may mean that students learn serendipitously with difficulty in ensuring a standardized learning experience. In an accreditation report, one anonymous student noted professional misconduct of her field educator who was behaving in a way that conflicted with what the student had learnt in class. The university teaching team was reported to have addressed the professional misconduct and related negative emotions with the student and turned these incidents into teachable moments. While education experience during WIL remained unstructured, it allowed students to gain insights into labor market expectations.

### Theme 3: prior experience, education interests, and team dynamics among students and teachers shaped learning and teaching experience

Human factors were found to contribute both positively and negatively to the learning and teaching experience. Human factors included the individual teacher’s passion and intrinsic interest in teaching, perceived commitment to education, resilience in dealing with complex student problems and heavy workload, teaching experience and peer support in the teaching team. Similarly, students were found to enter the learning process with different levels of education capacity and social skills, along with varied learning preferences and expectations. The learning culture within student cohorts and in-class peer interactions also varied from year to year. One teacher commented:

“…[t]he student-student relationship is so important. When they are in a group cohort within a class, with great dynamic, they learn well when they’re with their friends.” (Focus group 3, participant B)

A cohesive supportive culture within the student body was found to reduce anxiety in learning, however, this was often challenged by the diversity of the student cohort. The diversity in age, gender, culture, ethnicity, educational background, capacity and commitment could also result in a heavy workload experienced by teachers:

“…what we’re doing is herding cats! The huge diversity; where they [the students] come from in terms of countries and also whether [they have] previous training and also for them whether or not they are receiving and they are willing to learn and all that is really a big thing.” (Focus group 1, participant B)

Individual interpersonal relationships among staff in the teaching team and with WIL educators were reported to affect the quality of teaching. Some teachers purported to have reservations with specific WIL sites or a specific educator, but most teaching teams in the six programs described strategies to support novice WIL educators and offered to share workload within the teaching team when needed. Participants commented on important support strategies including strategies to identify struggling students, having clearly defined and detailed teaching materials, offering novice educators regular coaching by program coordinators, and site visits to WIL sites to support onsite educators and ensure clear communication of expectations. One participant noted:

“…[c]urrently we have our third years doing their placement [WIL] and we have lots of new preceptors [WIL educators]. We have been doing [a] lot of education for the preceptors so they understand how to assess and grade the students appropriately. Some also found us helping to bridge understanding between them and students, like highlighting they [students] don’t understand the scale [a clinical tool] because they haven’t learned it in class.” (Focus group 1, participant C)

During education interactions, teachers reported habitual collection of unstructured teaching feedback and informal evaluation of student assessment performance to inform modifications in teaching. Quality assurance feedback was mostly gathered in situations where the teachers were reflexive and had good relationships with students.

### Theme 4: professional identity provides structure for teachers and students

Professional identity and perceived professional obligations were reported to be significant drivers of behaviors among teachers, particularly the commitment to ‘pass down’ the knowledge and skills:

“…[i]t’s that complex arrangement for medicine where that’s the culture, and it comes down from consultants expected to facilitate learning for registrars, are expected to facilitate learning for interns, are expected to facilitate learning for, I don't know, whoever else… a lot of it’s to do with the history of how it developed and the roles of various levels of expertise in medicine and what they were supposed to do for the people following behind.” (Focus group 4, participant A)

Construction of a professional identity was embedded in the curriculum across the four vocational programs, with non-vocational health sciences programs the exception. The actions that a professional would take in practice were found to be used in teaching to anchor students’ understanding of what they need to be able to do at the end of the program while providing some contextual explanations of how the behavioral competencies are relevant in the profession. One participant noted:

“…[w]hen you’re looking at students who are coming through Medicine, Physio[therapy], Pharmacy, Nursing, Dietetics, they have a career that is at the end of that, which very much colours their process of learning, but also the process of teaching.” (Focus group 5, participant A)

Participants in the non-vocational degrees described the challenges in structuring the program and sparking inspiration among students when there is a lack of a clear job role at the end of the degree. One participant reported:

“I remember not knowing why I needed to do chemistry and biochemistry and everything and finding it so hard. And I still find it hard… because I’m just not going to engage with it because I don’t connect with it.” (Focus group 2, participant B)

### Multifactorial contribution to quality learning and teaching

**Figure 3:**
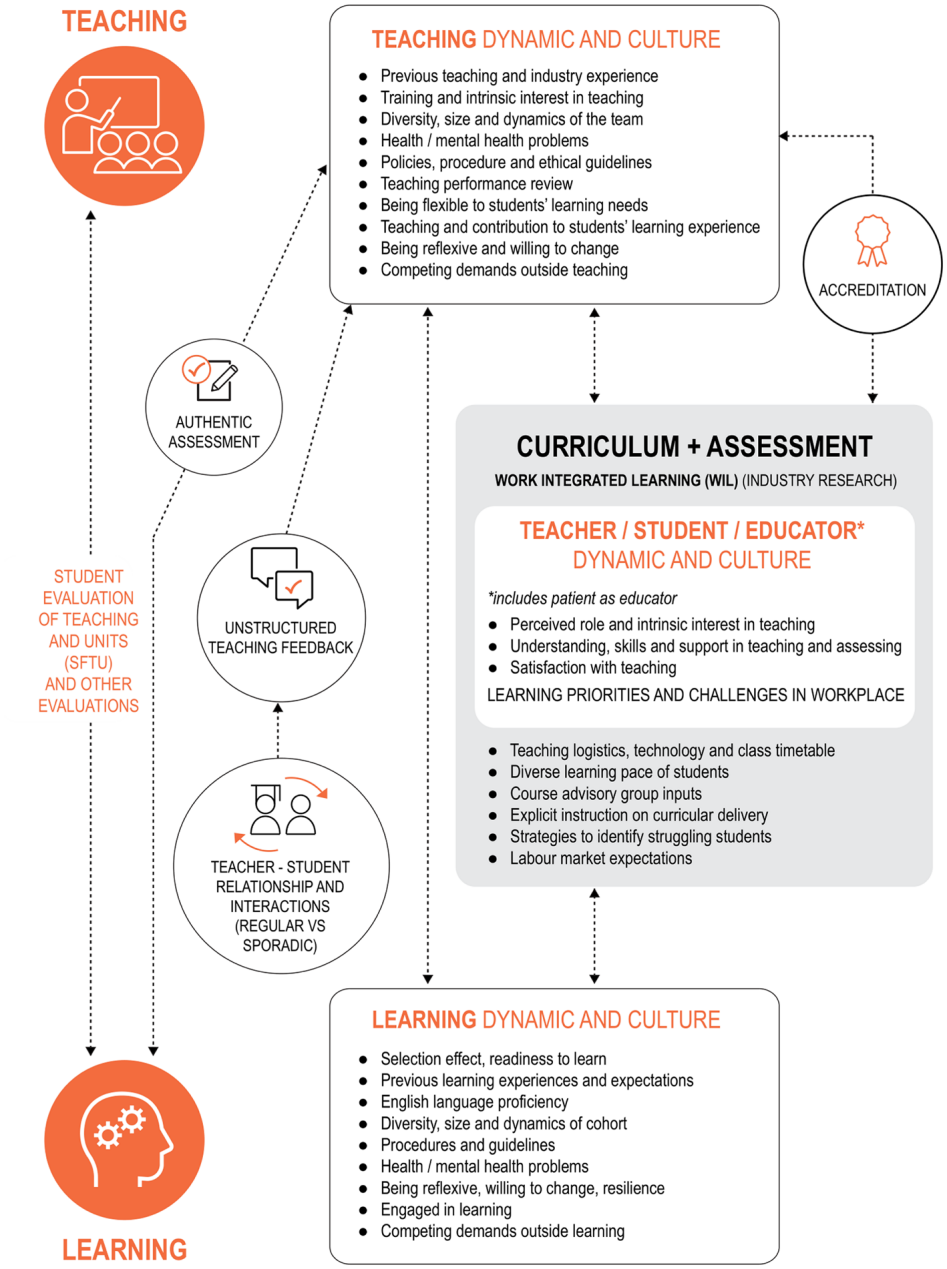
Contribution story of the proximal and distal factors influencing the achievement of graduate outcomes.

Based on the data, a Contribution Story was built to showcase the complex multifactorial influence on learning and teaching (Figure 3) between the six graduate outcomes and their causal properties. The existing data collected from document analysis and the narratives from the focus groups helped to unpack the three areas contributing to graduate outcomes identified as: (i) human factors (teacher related and student related), (ii) curricular factors (accreditation requirements, classroom and WIL, formal and informal feedback pathways), and (iii) organizational factors (teaching logistics, policies and procedures in place to guide and monitor teachers and students’ behaviors). A complex interplay between the above factors was identified alongside the constantly changing degree of influence on the quality and experience of learning and teaching. Moreover, the Contribution Story (Figure 3) showed that teacher dynamic and culture, student dynamic and culture and WIL context, all contributed to the development of graduates who (1) collaborate and work effectively in teams, (2) commit to lifelong learning, (3) demonstrate effective communication skills, (4) use and generate evidence; (5) improve health and (6) are professional in their practice. Teacher dynamic and culture, student dynamic and culture and WIL contexts were highlighted as factors that support and hinder teaching and learning, in addition to contributing to the development of competent health profession graduates.

## Discussion

Using CA as a novel approach, our study evaluated the six health professions and health sciences program and explored what factors support and hinder teaching and learning, and what teaching and learning factors contribute to the development of competent health profession graduates. Our analysis identified key variables involving students, teachers and a curriculum including WIL that affect the achievement of graduate outcomes. The four themes that emerged from our data demonstrated causal links among the curricula, the students and staff, and the development of desired graduate outcomes. We identified that strategies to facilitate desired graduate outcome development are often implied or ambiguous and could be interpreted differently by teachers. It is important for teachers to capitalize on episodic moments at WIL settings and to turn incidents into teachable moments and further enhance and consolidate the graduate outcome development. The team culture among teachers and students also contributes to creating a supportive environment for teaching and learning. Professional identity influences teaching practice and contextualizes expected professional behaviors for students. We captured the complex links between proximal and distal factors and the development of graduate outcomes and developed a contribution story, which could become a framework for future evaluation of health profession education programs.

Our analysis from document analysis and focus groups highlights that the target behavioral capabilities or competencies (e.g., oral communication, critical thinking, reflective practice, etc.) within curricular documents may not be explicitly articulated, and thus, affects the quality and efficiency, thus the outcomes of teaching and learning. This is not surprising, given recent work that has identified health care graduates from the same institution have multiple conceptualizations of preparedness for practice (26). These capabilities may not be taught in overt and transparent ways, that is either central to curriculum or valued through assessment practices. Such practice could have contributed to several implications in teaching, learning and assessment for both students and teachers.

It is well recognized that assessment drives learning, and students tend to prioritize a learning experience if it is linked to assessment (27, 28). When particular capabilities are not assessed explicitly, students may miss them or perceive them as less important. For many academics, especially sessional teachers, content-heavy courses coupled with large class sizes and limited contact time may impact on the pedagogical opportunities for incorporating behavioral capabilities into their teaching. It is important for courses, especially non-vocational courses, to establish a structured framework explicitly mapping the planned desired competency development into each assessment and activity.

Within the Contribution Story, we highlighted the significance of acknowledging the complex interconnected multifactorial relationship between curricular activities, learning cultures, WIL and development of desired graduate outcomes. In a health-based practice setting, WIL provides a platform for students to begin to think, act and feel like a health professional (29). Students in this environment are ideally exposed to rich learning experiences encompassing authentic problem-solving related to health-related issues that promote their intellectual capacity to apply conceptual, procedural and dispositional knowledge to real healthcare settings (29). Other benefits of WIL include students developing professional identities, working in interdisciplinary teams, having smoother professional transitions due to consumer exposure and developing skills of adaptability to face the rapidly changing labor market (30, 31).

Given that WIL is characterized by context-dependent, highly complex and multifaceted pedagogical approaches (32), it may increase variability in learning experiences for students. Our analysis revealed that student learning during WIL was opportunistic and incidental. This practice challenged some students to utilize the WIL experience to enhance their professional learning or gain insights into professional expectations. While ensuring that a consistent WIL experience for every learner is not feasible and may lose authenticity, future strategies are required to support WIL educators to capitalize on opportunistic teachable moments in workplace settings.

This study reinforced the need for shifting the course evaluation focus from narrow student satisfaction measures to more detailed and nuanced evaluation measures. Other studies have challenged the reification of student satisfaction surveys arguing that they are unable to fully capture the breadth of influences on teaching quality and outcomes (33). Our final Contribution Story further highlights that current student satisfaction measures alone are inadequate to capture the complexities of teaching and learning in health professions education.

With its range and diversity of factors supporting and hindering optimal teaching and learning experience in health profession education, CA allowed us to adopt a systematic approach to evaluate teaching quality. CA provided the opportunity to acknowledge the complexity of the teaching and learning process, permitting us to make credible causal claims to link learning and teaching activities to learner development of graduate outcomes.

### Strengths and limitations

The limitation of this study is the single Australian institution focus limiting the transferability of the findings to other institutions and countries. However, the representation of health profession and health science programs and breadth of data from documents and the heterogeneity of focus group participants in the analysis enhanced transferability of the findings. We acknowledge that the degree of influence by each factor in our Contribution Story on the development of graduate outcomes has not been measured because components intersect and interact in different combinations both contextually and temporally. We also acknowledge that our contribution analysis was built iteratively from one program to the next, and did not include examining the individual’s influence on producing graduate outcomes (i.e., how an outcome was attributed to individuals) or profession-specific cultural norms, professional identity or profession history. However, concurring with Schumacher et al. (34), we argue that the process of CA can be used to analyze specific components of individual’s behavior and practice attributed to achieving specific graduate outcomes. This application of CA enhances the scope for future research employing both contribution and attribution analysis in evaluating education programs. The choice of using the CA approach, a theory-based, impact evaluation method with the rigor applied to data analysis and involvement of the large and diverse research team further strengthened the study’s interpretations and findings. Future research should consider testing the variables identified in this study as markers of education outcomes across other contexts.

## Conclusion

Our study presents CA as a theory-based evaluation approach of health professions education, exploring multiple data sources as evidence and acknowledging the complexity of human, curricular and organizational factors toward high quality teaching. This Contribution Story also identifies teacher and student dynamics and cultures, and WIL contexts as important variables for evaluation of health professions education and provides a framework that can be used by others planning meaningful course evaluations within health education programs and beyond.

## Data availability statement

The datasets presented in this article are not readily available because the data is not available due to the nature of the data and ethics requirements. Requests to access the datasets should be directed to TC, tammie.choi@monash.edu.

## Ethics statement

The studies involving humans were approved by Monash University Human Ethics Committee. The studies were conducted in accordance with the local legislation and institutional requirements. The participants provided their written informed consent to participate in this study. Written informed consent was obtained from the individual(s) for the publication of any potentially identifiable images or data included in this article.

## Author contributions

CP and MSa: study conception and design. TC and MSa: data collection, analysis, and interpretation of results. TC, MSa, and CP: draft manuscript preparation. All authors reviewed the results and approved the final version of the manuscript.

## Funding

This work was supported by the Monash University Faculty of Medicine, Nursing and Health Sciences Learning and Teaching Large Grant.

## Conflict of interest

The authors declare that the research was conducted in the absence of any commercial or financial relationships that could be construed as a potential conflict of interest.

## Publisher’s note

All claims expressed in this article are solely those of the authors and do not necessarily represent those of their affiliated organizations, or those of the publisher, the editors and the reviewers. Any product that may be evaluated in this article, or claim that may be made by its manufacturer, is not guaranteed or endorsed by the publisher.
